# Supplementary material for: Anti-platelet factor 4/polyanion antibodies mediate a new mechanism of autoimmunity
Source: Nat Commun. 2017 May 22;8:14945. doi: 10.1038/ncomms14945 (PMC5458132; doi:10.1038/ncomms14945)
Supplement: Supplementary Information — Supplementary Figures and Supplementary Table. [file ncomms14945-s1.pdf]

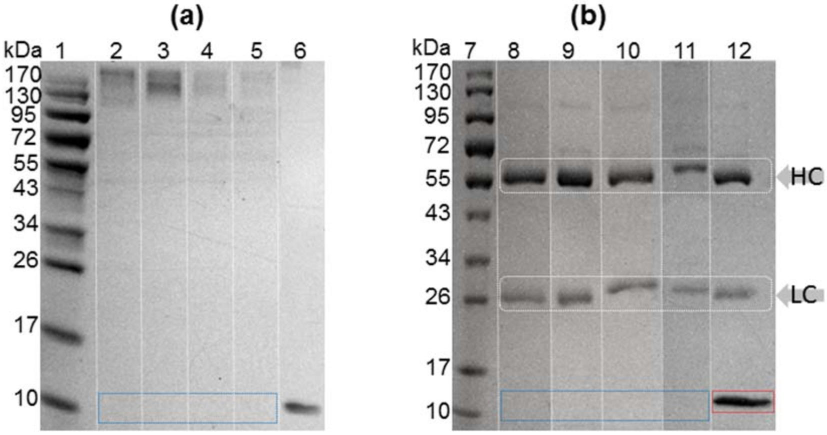

Supplementary Figure 1. SDS-PAGE of purified PF4/H ABS concentrated to 100 µg/ml

(**a**) non-reducing conditions: lane 1: marker; lane 2: control IgG; lane 3: group-1 ABS; lane 4: group-2 ABS; lane 5: group-3 ABS; lane 6: purified PF4; (**b**) reducing conditions: lane 7: marker; lane 8: control IgG; lane 9: group-1 ABS; lane 10: group-2 ABS; lane 11: group-3 ABS; and lane 12: purified anti-PF4/H ABS contaminated with PF4 releasing from column. Markers (lane 1 and 7) with molecular weight up to 170 kDa were used. Molecular weights of ABS under non-reducing condition are ~170 kDa; IgG was split into heavy chains (HC; upper white rectangular) and light chains (LC; lower white rectangular) under reducing condition. Only samples which were free of PF4 (blue rectangles) were used. If PF4 was found in the purified ABS fraction (red rectangular; lane 12); the ABS fraction was re-purified using a protein G column (the figure shows representative examples of n=5 antibody fractions analyzed for each antibody group).

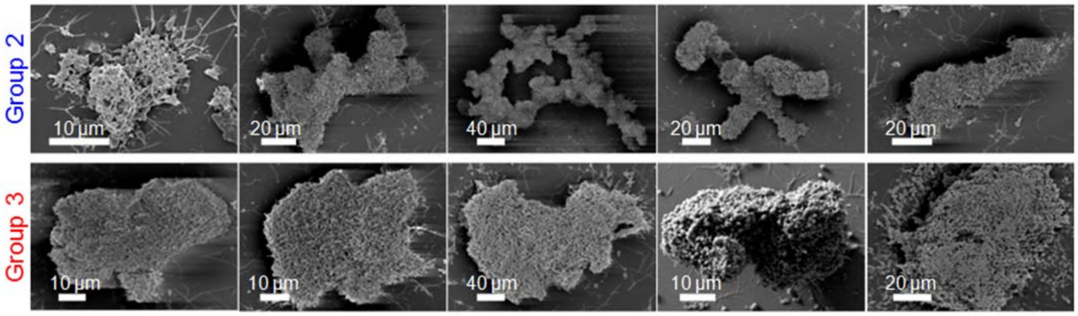

Supplementary Figure 2. SEM images of platelet aggregates in the HIPA test in the presence of reviparin after 15 min mixing

Platelets formed with group-2 ABS relaxed and extended clusters with many branches and tails (upper row), but tightly packed clusters in the presence of the group-3 ABS (bottom row). Scale bars are given in each image.

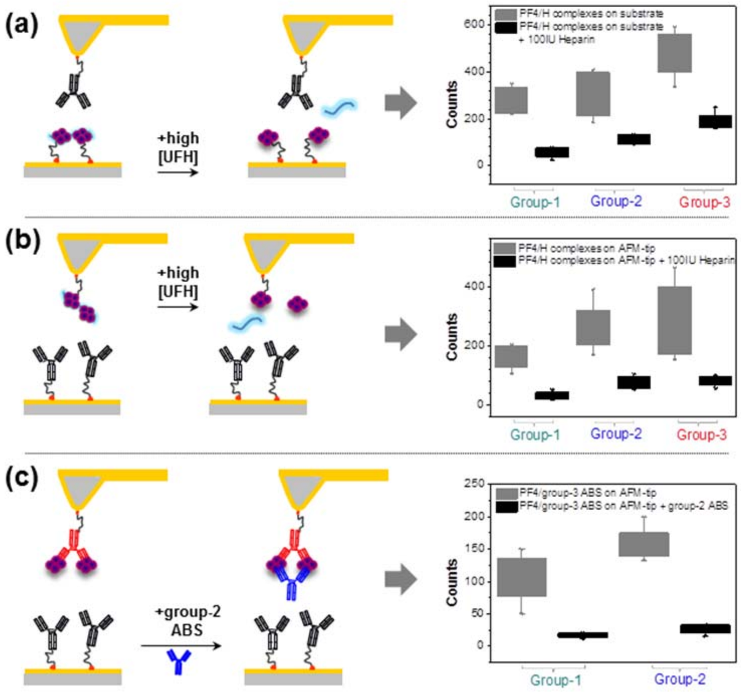

Supplementary Figure 3. Blocking experiments

(a) PF4/H complexes coated on the substrate are disrupted in the presence of high heparin concentrations (100 IU/mL) resulting in massive reduction of interaction events (counts). (b) The interaction reduced for all antibodies when they were incubated with PF4/H complexes on the tips in the presence of high heparin concentrations (100 IU/mL). (c) Blocking of binding sites on PF4/group-3 ABS complexes on the tip by group-2 ABS results in the minimal binding events with immobilized group-1 and group-2 ABS on the solid phase.

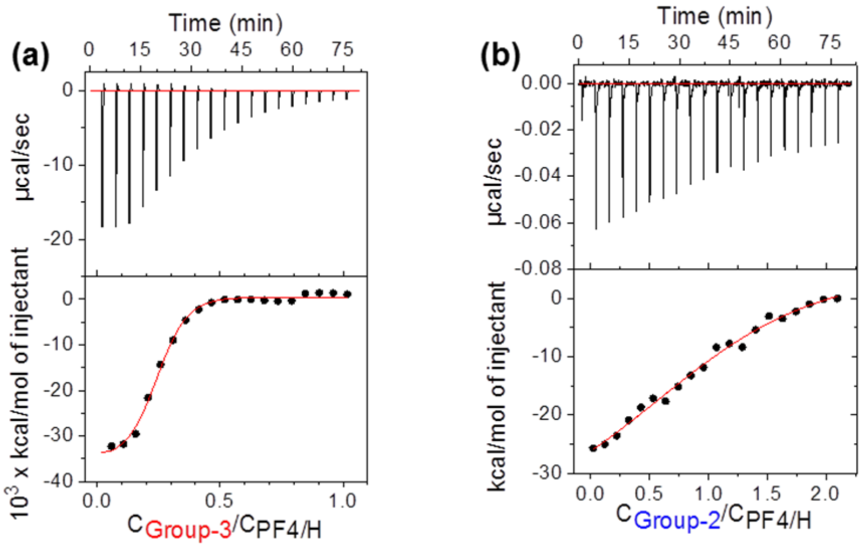

(c)

Purified anti-PF4/P-ABS		Stoichiometry, N (sites)	K_D (nM)	ΔH (cal/mol)	ΔS (cal/mol/K)
With PF4/H complexes	Group-2 (n = 2)	0.37 ± 0.03	1.7 x 10^2 ± 0.6	-2.9 ± 0.40 x 10^4	-66.3 ± 14.6 x 10^0
	Group-3 (n = 2)	0.27 ± 0.04	5.3 ± 0.5	-2.9 ± 2.06 x 10^8	-90.1 ± 69.0 x 10^6
With PF4	Group-3 (n = 2)	0.53 ± 0.003	1.8 ± 1.3	-3.5 ± 0.86 x 10^7	-11.7 ± 2.8 x 10^4

Supplementary Figure 4. Thermodynamics of the interaction of group-2 and group-3 ABS with PF4/H complexes or PF4 obtained by Isothermal Titration Calorimetry (ITC)

Representative binding isotherms for the titration of (**a**) group-2 or (**b**) group-3 ABS into the sample cells containing PF4/H complexes: raw titration data (upper panels), and integrated heats (lower panels). At concentrations of 62.5 nM, the thermograms clearly changed for both antibody groups (n = 2). **(c)** Thermodynamic parameters of the interactions. The interaction between group-3 ABS and PF4 (ΔH = -3.5 ± 0.86 x 10^7 cal/mol) or PF4/H complexes (ΔH = -2.9 ± 2.06 x 10^8 cal/mol) released much higher heat than that of the group-2 ABS (ΔH = -2.9 ± 0.4 x 10^4 cal/mol). The lower dissociation constant (K_D) ~30-fold indicates that group-3 ABS formed with PF4 (K_D = 1.8 ± 1.3) or PF4/H complexes (K_D = 5.3 ± 0.5) much more stable complexes than group-2 ABS (K_D = 170 ± 0.6). Stoichiometry (n: binding sites) also indicates that group-3 ABS cluster two PF4 tetramers (N = 0.53). Standard deviation '±' was obtained from antibodies purified from two sera per group.

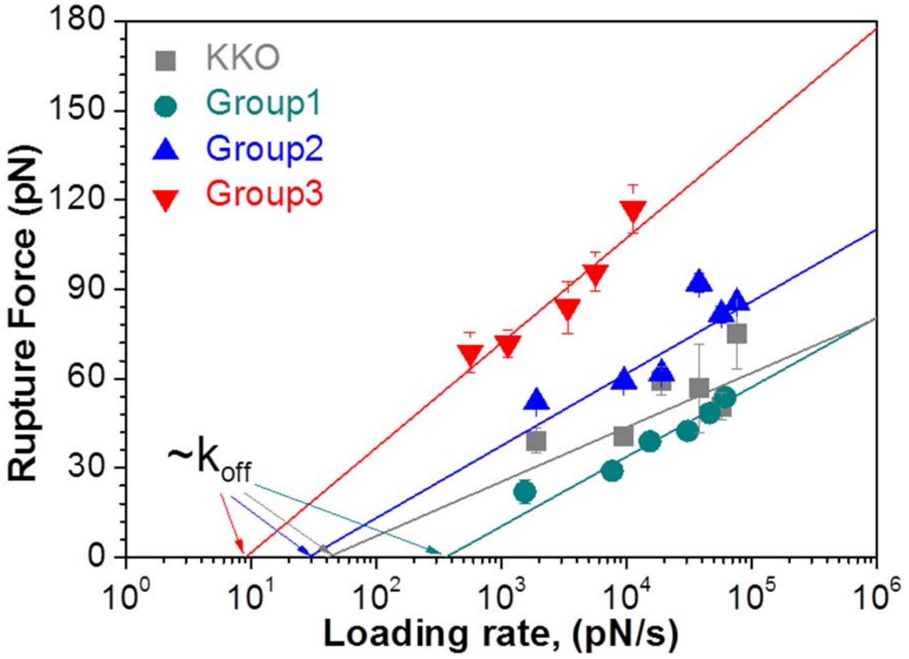

Supplementary Figure 5. Binding kinetics of antibodies to PF4/H complexes

Rupture forces recorded at different loading rates for KKO (grey), group-1 (dark cyan, n = 3), group-2 (blue, n = 3) and group-3 ABS (red, n = 3). Group-1 ABS show highest k_{off} value (15.6 s^{-1}), while KKO (2.2 s^{-1}) has a similar thermal off-rate as group-2 ABS (2.0 s^{-1}), and group-3 ABS exhibit the lowest k_{off} value (0.12 s^{-1}). The results indicate that group-3 ABS have the highest binding affinity to PF4/H complexes, followed by KKO and group-2 ABS, while group-1 ABS have the lowest binding affinity. KKO and group-1 samples contain homogeneous antibodies, while group-2 and group-3 contain antibodies with different characteristics. We therefore, pre-tested the cantilevers coated with either group-2 or group-3 ABS and selected cantilevers which had an antibody bound, showing an interaction force ~70 pN for group-2 or ~90 pN for group-3 ABS for measurements. Since these antibodies were preselected, we did not observe the large variation of rupture force distributions compared to the analysis of the unselected PF4/H ABS fraction shown in Fig. 3 and Fig. 4 (main text). Data were obtained from the mean values of different antibodies obtained from different sera (n=3).

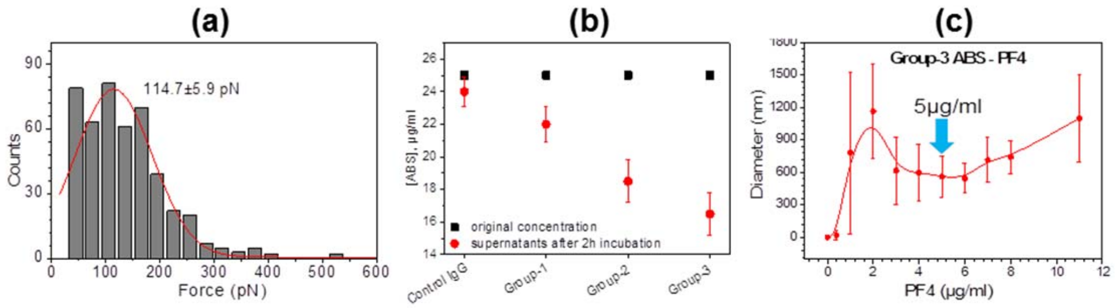

Supplementary Figure 6. Additional evidence that group-3 ABS cluster PF4

(a) ABS purified by a PF4 column and coated to the cantilevers interact with PF4/H complexes showing a mean binding force (± the associated mean error) of 114 ± 5.9 pN (n=3). (b) PF4/group-3 ABS complexes immobilized on the substrate created a target recognized by group 1-3 ABS. Different ABS (n=2 for each group of ABS; 25 µg/mL) were incubated with PF4/group-3 ABS complexes coated on the substrates. After 2h incubation, the ABS concentration of the supernatants (red) reduced slightly for group-1 ABS, stronger for group-2 and strongest for group-3 ABS but no significant change was observed for control IgG. (c) At PF4 concentrations ≤3 µg/ml, group-3 ABS (10 µg/ml) form unstable complexes with PF4 as indicated by a large variation in size, stable complexes were formed at PF4 concentrations from 3 to 6 µg/ml, and larger complexes were formed at PF4 concentrations ≥7 µg/ml. A PF4 concentration of 5µg/ml was selected for incubation with group-3 ABS (10 µg/ml) to subsequently test the interaction of these complexes with group-1 and group-2 ABS shown in Fig 5g of the main text.

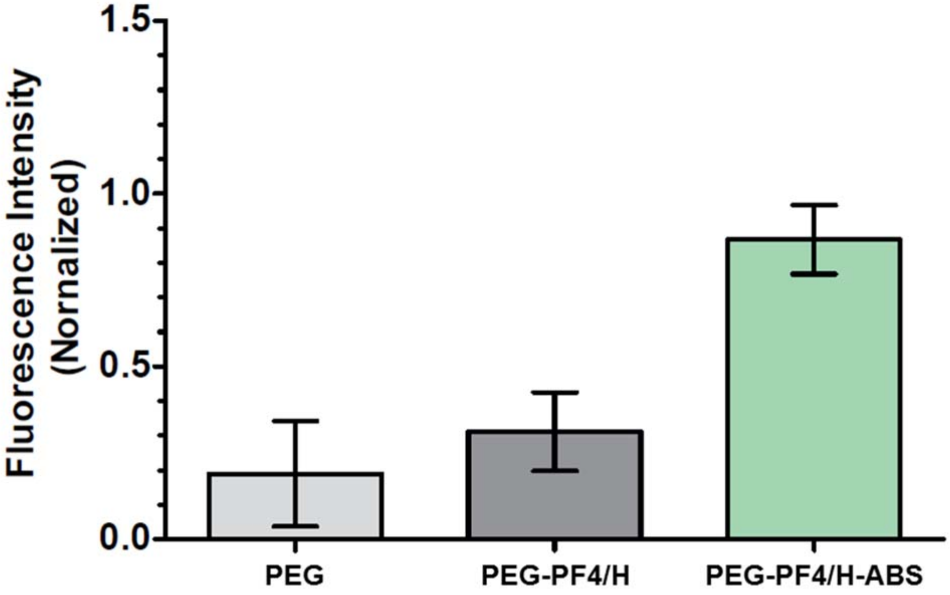

Supplementary Figure 7. Quality control of surface coating for SMFS experiments

Binding of PF4/H ABS to the coated PF4/H complexes was tested by fluorescent labeled anti-human IgG antibodies. Surface coated with PEG alone (light gray) shows lowest fluorescence intensity, higher fluorescence was obtained when PF4/H complexes bound to PEG (dark gray column; background signal of the experiment), and the highest signal was obtained when PF4/H ABS bound to PF4/H complexes immobilized on PEG (green). These results indicate that the antigenic complexes were maintained after binding to the solid phase**.**

Supplementary Table 1. Characteristics of sera and purified PF4/P ABS

Antibody group	Sera	PF4/P IgG-EIA (OD_{450nm})		HIPA					
				Serum			Purified ABS		
		Original serum (1:200)	Purified ABS (20 µg/mL)	Buffer	Reviparin 0.2 IU/mL	Heparin 100 IU/mL	Buffer	Reviparin 0.2 IU/mL	Heparin 100 IU/mL
**Group-1 (G1)** n = 5	G1-1	2.06	0.76	-	-	-	-	-	-
	G1-2	1.78	1.20						
	G1-3	1.72	0.59						
	G1-4	1.86	1.17						
	G1-5	2.01	1.50						
**Group-2 (G2)** n = 5	G2-1	2.40	1.37	-	+	-	-	+	-
	G2-2	2.30	1.99						
	G2-3	1.29	1.81						
	G2-4	2.06	1.71						
	G2-5	2.6	2.26						
**Group-3 (G3)** n = 5	G3-1	2.68	3.27	+	+	-	+	+	-
	G3-2	2.40	1.52						
	G3-3	2.82	2.74						
	G3-4	2.29	3.17						
	G3-5	2.60	1.81						

PF4 = platelet factor 4; P = polyanion; ABS = antibodies; OD = optical density; IU = international units

EIA and HIPA characteristics of the three antibody groups (n=5 sera/group). The purified antibodies showed the same characteristics as their original sera in the HIPA test. The lower optical density (OD) values in the PF4/heparin (PF4/H) EIA for the purified group-1 ABS indicate that these antibodies might be present in serum at concentrations above 20 µg/mL.
